# When are patients lost to follow-up in pre-antiretroviral therapy care? a retrospective assessment of patients in an Ethiopian rural hospital

**DOI:** 10.1186/s40249-015-0056-y

**Published:** 2015-06-01

**Authors:** Tamrat Shaweno, Debebe Shaweno

**Affiliations:** Public Health Emergency Response Epidemiologist, African Union, Addis Ababa, Ethiopia; School of Public and Environmental Health, College of Medicine and Health Sciences, Hawassa University, Hawassa, Ethiopia

**Keywords:** Pre-ART, Loss to follow-up, Rural Ethiopia

## Abstract

**Background:**

There is concern about the increasing rates of loss to follow-up (LTFU) among pre-antiretroviral therapy (pre-ART) patients in Ethiopia. Little information is available regarding the time when pre-ART patients are lost to follow-up in the country. This study assessed the time when LTFU occurs as well as the associated factors among adults enrolled in pre-ART care in an Ethiopian rural hospital.

**Methods:**

Data of all adult pre-ART patients enrolled at the Sheka Zonal Hospital between 2010 and 2013 were reviewed. Patients were considered lost to follow-up if they failed to keep scheduled appointments for more than 90 days. The Cox proportional hazards regression model was used to assess factors associated with time until LTFU. The Kaplan-Meier survival table was used to compare the LTFU experiences of patients, segregated by significant predictors.

**Results:**

A total of 626 pre-ART patients were followed for 319.92 person-years of observation (PYOs) from enrolment to pre-ART outcomes, with an overall LTFU rate of 55.8 per 100 PYOs. A total of 178 (28.4%) pre-ART patients were lost to follow-up, 93% of which occurred within the first six months. The median follow-up time was 6.13 months. The independent predictors included: not having been started on co-trimoxazole prophylaxis (adjusted hazard ratio [AHR] = 1.77, 95% confidence interval [CI], 1.12–2.79), a baseline CD4 count of or above 350 cells/mm3 (AHR = 1.87, 95%CI, 1.02–3.45), and an undisclosed HIV status (AHR = 3.04, 95%CI, 2.07–4.45).

**Conclusion:**

A significant proportion of pre-ART patients is lost to follow-up. Not having been started on co-trimoxazole prophylaxis, presenting to care with a baseline CD4 cell count ≥350 cells/mm^3^, and an undisclosed HIV status were significant predictors of LTFU among pre-ART patients. Thus, close monitoring and tracking of patients during this period is highly recommended. Those patients with identified risk factors deserve special attention.

**Electronic supplementary material:**

The online version of this article (doi:10.1186/s40249-015-0056-y) contains supplementary material, which is available to authorized users.

## Multilingual abstracts

Please see Additional file 1 for translations of the abstract into the six official working languages of the United Nations.

## Background

At the end of 2013, 35 million people were living with HIV worldwide, of which nearly 10 million had started antiretroviral therapy (ART) [1,2]. More than two-thirds of the global cases are located in Sub-Saharan Africa (SSA) [2-5]. Not all patients who present at an earlier stage of their illness are eligible for ART, and even if they are eligible, prompt initiation of treatment will depend on several factors including availability of medicines and trained health workers [3,6,7]. Eligible patients are also hindered from accessing heath care and initiating treatment due to factors such as distance to healthcare facility, social stigma, being in a less advanced disease stage, feeling healthy, occupation, and level of literacy [2,4,6,8-11]. It is therefore likely that in settings with a high disease burden and limited resources, some patients will default from care before they are started on ART [3,4]. A review of literature on patient retention in SSA showed that less than one-third of patients remain continuously in care between the time they test HIV-positive and start ART [12].

Loss to follow-up [LTFU) among patients enrolled into HIV care is a persistent challenge in SSA. Although LTFU among pre-ART patients poses challenges in Ethiopian ART hospitals [3,6,12-14], it has only recently began to be recognized in literature [12]. High pre-ART patient loss is a signal of poor uptake of interventions. Historically, strategies to address patient LTFU have focused on the ART period, with greater attention and resource prioritization given to this, however, LTFU among pre-ART patients has been given less attention [15-19].

The facility-based annual report of HIV progress at the Sheka Zonal Hospital indicated that a significant number of patients who tested positive for HIV failed to start pre-ART care and those linked to care were lost before ART initiation [20]. A better understanding of the time when patients are lost to follow-up and associated risk factors in rural Ethiopia could be helpful for designing time relevant intervention strategies. The aim of this study was to assess the time when patients are lost to follow-up and the associated factors in a cohort of pre-ART patients at the Sheka Zonal Hospital, rural Ethiopia.

## Methods

### Study setting and design

This was a retrospective assessment of patients enrolled in pre-ART care from 2010 to 2013 at the Sheka Zonal Hospital, southwest Ethiopia, which is located about 611 km from the capital, Addis Ababa. The estimated catchment population was 120,000, sub-divided into 19 rural and three urban kebeles, of which seven rural and three urban were located within 10 km of the hospital, the recommended distance for service accessibility [20,21]. The institution started providing ART in 2006. Other services including voluntary counseling and testing (VCT), provider-initiated counseling and testing (PICT), prevention of mother-to-child transmission (PMTCT), and condom promotion are delivered integrally. Patients are linked to pre-ART care on the same day of testing by using both internal and external modes of patient referral systems. Linked patients are continuously followed-up on pre-ART care until they are eligible to start lifelong antiretroviral drugs (ARVs). The CD4 measurement of pre-ART patients is taken on the same day of testing for HIV and then every six months (on average). All patient information is registered and kept confidentially both in hard and electronic formats. Pre-ART patients are appointed for care and follow-up every three months as per the national and World Health Organization (WHO) guidelines, except for those in clinical urgency and chronic management. Adherence supporters and case managers collect patient information. All patient-related data are coded, entered, and analyzed by data clerks trained by management science for health (MSH) and John Hopikin’s university (JHU) TSEHAI. Up to the time of data collection, 2,170 patients have been enrolled in pre-ART care since the service started.

### Sample size

All 626 adult HIV positive patients enrolled in pre-ART care at the Sheka Zonal Hospital from September 1, 2010 to August 31, 2013 were included in the study.

### Variables

In this study, the baseline explanatory variables were age, sex, pregnancy status, religion, educational status, marital status, number of children, dependent children in the household, place of residence, occupation, disclosure of HIV status, number of CD4 cells count, WHO clinical stage, provision of co-trimoxazole prophylaxis therapy (CPT), opportunistic infections, TB status, BMI, baseline functional status of the patient, availability of ART clinic in the catchment area (<10 km), and mode of patient referral system. The major pre-ART outcome was time until LTFU, defined as the time from the date of enrolment up to 90 days after the last scheduled appointment [16,22]. Those who came back after 90 days of the last scheduled appointment were treated as LTFU cases. On the other hand, those who were active on follow-up, were transferred to other facilities, or initiated treatment at the end of the observation period were reported as censored cases.

Data were extracted both from the electronic database and standard registries using a checklist developed from the WHO and Ethiopian national treatment guidelines for pre-ART care [13,23].

### Data processing, analysis, and interpretation

Data coding, entry, and cleaning was performed using EpiData version 3.1 and analysis was done using SPSS version 16.0. To investigate predictors of timing until LTFU, two strategies were used. First, explanatory variables were entered into separate bivariate Cox regression proportional hazards models. Second, all variables from the bivariate models with *p*-value ≤0.25 were included in a final multivariable Cox regression model. Associations were examined at a *p* <0.05 significance level.

Rates of LTFU were calculated by summing the number of patients lost to follow-up during a particular period of time divided by the total number of years of follow-up during this period. The proportional hazard assumption was assessed by performing log-log survival curves based on Schoenfeld residuals. Variables with incomplete data were entered as “missing”; the rate of missing data for all variables was below 2.7%.

The Kaplan-Meier survival table was used to compare LTFU experiences of pre-ART patients segregated by significant predictor variables at enrolment. Incidence density was calculated for LTFU cases using person-years of contribution to the cohort.

### Ethical statement

The study protocol was approved by the Institutional Review Board of Jimma University, Ethiopia.

## Results

Between 2010 and 2013, a total of 659 patients were enrolled in pre-ART care. Of these, 626 were eligible for this study based on the inclusion criteria.

### Socio-demographic characteristics of the patients

The mean age of participants was 30.6 (SD, 8.7) years, 59.4% were females, and 4.4% of the females were pregnant. Of the total participants, 58.0% were married, 34.4% had dependent children below 15 years of age, 39.5% had primary level education, and 67.3% were Orthodox Christians. More than half (50.8%) were from rural communities and 34.9% were of the Amhara ethnicity. The selected socio-demographic characteristics of the study participants, by pre-ART outcome, are presented in Table 1.

**Table 1:** **Baseline socio-demographic characteristics of adult pre-ART patients enrolled in pre-ART care at the Sheka Zonal Hospital, Sheka Zone, southwest Ethiopia, 2010–2013, by pre-ART outcome**

**Socio-demographic variable**		**LTFU n (%)**	**Retained n (%)**
***Age (in years)***	15–24	58 (32.6)	87 (22.0)
	25–34	80 (44.9)	190 (48.1)
	35–44	30 (16.9)	84 (21.3)
	44+	10 (5.6)	34 (8.6)
***Sex***	Male	67 (37.6)	158 (40.0)
	Female	111 (62.4)	237 (60.0)
***Occupation***	Employed	40 (22.5)	71 (18.0)
	Unemployed	67 (37.6)	175 (44.3)
	Merchant	23 (12.9)	76 (19.2)
	Other	48 (26.9)	73 (18.5)
***Educational status***	No education	18 (10.1)	72 (18.2)
	Primary	68 (38.2)	160 (40.5)
	Secondary	51 (28.7)	87 (22.0)
	Tertiary and above	41 (23.0)	76 (19.2)
***Religion***	Orthodox	121 (68.0)	273 (69.1)
	Muslim	30 (16.9)	57 (14.4)
	Protestant	23 (12.9)	55 (13.9)
	Other	4 (2.2)	10 (2.5)

### Baseline characteristics of the patients

Of the 626 patients enrolled in pre-ART care, 374 (59.7%) were early stage presenters and 413 (66%) were linked to care with a baseline CD4 count ≥350 cells/ml. The baseline mean CD4 cell counts were 469.9 (SD, 323.2) cells/mm^3^ and 879.7 (SD, 349.5) cells/mm^3^for retained and LTFU patients, respectively.

Of the 79.2% of patients who had experienced at least one type of opportunistic infection during presentation to care, 341 (54.5%) had been treated with CPT.

Of the total participants, 227 (37.2%) were underweight (BMI ≤18.5), 377 (61.8%) were of normal weight (BMI = 18.5–24.9), and 6 (1.0%) were overweight (BMI ≥25). Regarding tuberculosis (TB) screening, 625 (99.8%) were screened for TB, of which 106 (17%) were positive.

In terms of the baseline functional status of the cohort during presentation to care, 578 (92.6%) were at working conditions and the rest (4%) and (3.4%) were at ambulatory and bedridden conditions, respectively.

### Service availability, mode of referral, and disclosure of HIV status

Of the total participants, 424 (67.9%) were in the catchment area of the zonal hospital and 528 (85.2%) were linked to pre-ART care using the internal mode referral. Regarding disclosure of HIV status, 368 (59%) disclosed their HIV status, of which 332 (90.2%) did so to their family members. The rest of the patients (41%) had not disclosed the status to anyone.

### Outcomes during follow-up period

Patients were followed up with pre-ART care from 2010 to 2013 for 319.92 person-years of observation (PYOs). At the end of the observation period, of the patients who were enrolled in pre-ART care, 178 (28.4%) were lost to follow-up, 20 (3.2%) were transferred to another facility, 268 (42.8%) had initiated treatment, 33 (5.3%) had died, and 127 (20.3%) were found to be active in pre-ART care. The overall LTFU rate was 55.8 per 100 PYOs. Among those patients lost to follow-up, 111 (62.4%) were female. The number of LTFU cases among pregnant women in the pre-ART patient cohort was not reported during the study period.

### Survival analysis of time until LTFU

A total of 626 patients were followed for a median of 6.13 (IQR, 4.57–13.23) months in pre-ART outcomes. The minimum follow-up time was three days and the maximum was 1,080 days (around 36 months). After a six-month period, 166 (93.26%) patients were lost to follow-up and this number grew to 171 (96.07%) at the end of one year, starting from the period of enrolment (see Table 2).

**Table 2:** **Estimated number of LTFU cases among pre-ART patients at the end of observation periods spanning 3, 6, 12, 24, and 36 months at Sheka Zonal Hospital, southwest Ethiopia, between 2010 and 2013**

**Observation period (in months)**	**LTFU cases**	**Cumulative loss**	**Cumulative loss in %**
***At 3 months***	136	136	76.40
***At 6 months***	30	166	93.26
***At 12 months***	5	171	96.06
***At 24 months***	7	178	100.00
***At 36 months***	0	178	100.00
**Total**	178		

The mean estimate of LTFU time for patients who started CPT at pre-ART care during enrolment was 9.88 (95% CI, 9.5–8.3) months, whereas for patients who were not started on CPT, it was 5.55 (95% CI, 4.9–6.2) months. The difference in time between the two categories of patients was statistically significant (*p* <0.001). Similarly, the mean estimate of time until LTFU for patients who disclosed their HIV status to their family members was 9.8 (95% CI, 9.4–10.3) months, whereas for patients who did not disclose their HIV status to anyone when enrolling into care was 5.0 (95% CI, 4.3–5.7) months. The observed difference was statistically significant (*p* <0.001).

At enrolment, the cumulative proportions of patients retained were higher among patients who started CPT, disclosed their HIV status to their family members, and who had a baseline CD4 ≥ 350 (see Table 3).

**Table 3:** **Comparisons of LTFU experiences among pre-ART patients, by CPT status and HIV status disclosure at enrolment, Kaplan-Meier survival table**

**Baseline variable**	**Time in months**	**Status**	**Cumulative proportion surviving at time T**	**N of cumulative attritions**	**N of remaining cases**
***CPT provision***					
Yes	At 3	LTFU	0.93	20	316
	At 6	LTFU	0.88	30	102
No	At 3	LTFU	0.61	111	117
	At 6	LTFU	0.49	136	108
***Disclosure of HIV status***					
To family	At 3	LTFU	0.93	23	309
	At 6	LTFU	0.89	29	119
Not at all	At 3	LTFU	0.59	105	151
	At 6	LTFU	0.46	125	74

CPT: co-trimoxazole prophylaxis therapy.

### Factors associated with time to LTFU

During the bivariate analysis of factors associated with time to LTFU, six explanatory variables with *p*-values <0.25 were selected for the multivariate Cox regression model. These included provision of CPT at enrolment, service availability, CD4 cell count, opportunistic infections, WHO clinical stage, and HIV status disclosure. Three predictor variables, CPT at enrolment, baseline CD4 count, and HIV status disclosure, were found to be independent predictors of time until LTFU (see Table 4).

**Table 4:** **Cox regression analysis for independent predictors of timing until LTFU at the Sheka Zonal Hospital, southwest Ethiopia, from 2010 to 2013**

**Variables**	**Crude HR**	**95% CI**	***P*** **-value**	**AHR**	**95.0% CI AHR**	***P*** **-value**
***CPT at enrolment***						
Provided	1.0			1.0		
Not provided	3.02	2.04–4.48	0.000	1.77	1.12–2.79	0.015
***CD4 category***						
<350	1.0			1.0		
≥350	7.20	2.96–17.96	0.04	1.87	1.02–3.45	0.044
***Disclosure of HIV status***						
To family members				1.0		
To other relatives	2.43	1.47–2.66	0.008	2.0	1.03–3.90	0.044
No disclosure	4.05	2.86–5.72	0.000	3.04	2.07–4.45	0.000
***WHO clinical stage***						
I/II	1.55	1.02–2.36	0.038	0.99	0.65–1.53	0.99
III/IV	1.0			1.0		
***Service availability***						
Within <10 km	1.0			1.0		
≥10 km	1.92	1.43–2.58	0.000	1.28	0.93–1.74	0.13
***Opportunistic infections***						
Yes**	1.0			1.0		
No	1.93	1.44–2.59	0.000	1.24	0.89–1.72	0.198

**Reference category, CPT: co-trimoxazole prophylaxis therapy, AHR: adjusted hazard ratio, CI: confidence interval.

## Discussion

Currently, the need to focus on retaining patients starting pre-ART care and not just patients being initiated on ART is being emphasized [5,6,18,24,25]. This study examined LTFU among pre-ART patients and associated factors in rural Ethiopia. In this study, the rate of LTFU in pre-ART care was high and was significantly associated with not having been started on CPT, presenting to care with a baseline CD4 ≥ 350 cells/mm^3^, and an undisclosed HIV status.

In this study, the overall rate of LTFU in pre-ART care was 28.4%. Compared to findings from different settings, this percentage is higher [18,26,27]. The differences could be due to variations in study design, definitions, and patient follow-ups. Unlike our study, which defined pre-ART patients as all newly enrolled HIV patients regardless of their clinical stage and CD4 count, studies conducted in other settings [17,25,28] focused on pre-ART patients with low CD4 cell counts and an advanced disease stage.

The incidence of pre-ART LTFU was highest in the first year of study. Approximately 96% of LTFU cases occurred in the first year and all LTFU occurred at the end of the two-year observation period. This is higher by nearly 10% when compared to findings from Nigeria that showed that 88.0% of LTFU cases occurred in the first year and all deaths occurred in the first six months of pre-ART care [12]. This variation may be due to poor monitoring and tracking of defaulting patients observed during early periods of pre-ART care [6,12,16].

**Figure 1:**
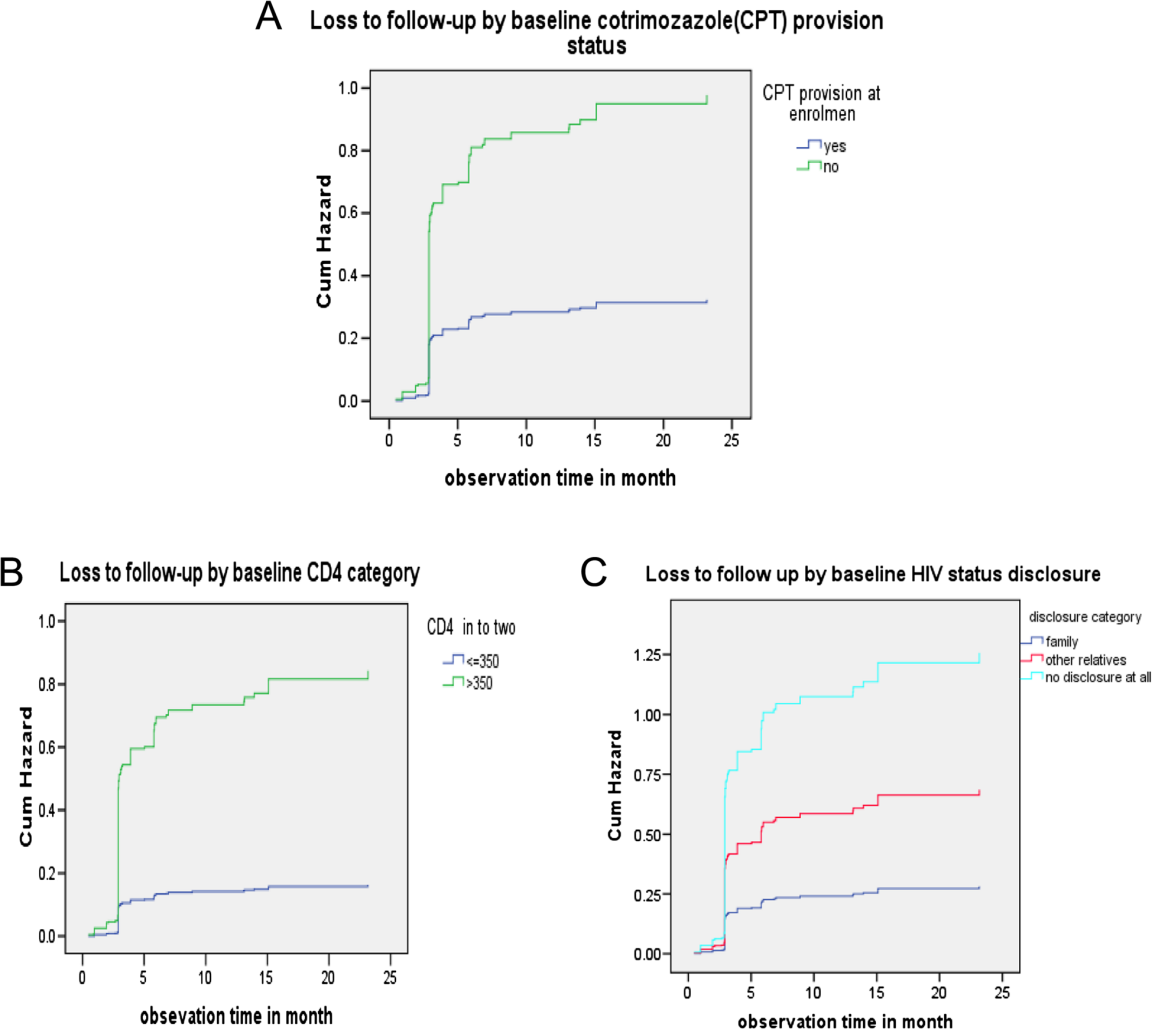
Pre-ART patient LTFU experiences segregated by baseline **(A)** Cotrimoxazole (CPT) provision **(B)** baseline CD4 category and **(C)** HIV disclosure status among adult pre-ART patients enrolled to pre-ART care at Sheka Zonal Hospital, rural Ethiopia, during 2010–2013.

Over three-quarters of pre-ART patients (83.8%) who did not start CPT at enrolment were lost to follow-up, compared to about 16% of those who did. The time until LTFU for patients who were started on CPT at pre-ART care enrolment was nearly two-fold higher than those who were not started on CPT. Furthermore, the patients on CPT had more than a 30% reduction in risk of LTFU six months into treatment and an increased retention time after adjustment for other explanatory variables compared to those who did not start CPT at enrolment, with the difference statistically significant (see Figure 1A). In a Kenyan study, treatment of ART ineligible patients with co-trimoxazole improved the 12-month retention in care from 63% to 84%; this suggests that patients may have perceived more benefit from CPT. Part of the underlying reasons for the high rate of LTFU among pre-ART patients not treated with CPT could be the lack of means for engaging these patients in care. Such patients are often not treated with CPT for opportunistic infections when their CD4 cell counts are high or if patients are at less advanced disease stages and it is indicated that provision of free medicines or nutrition can improve visits to the clinic and therefore, the retention in pre-ART care [6,12,29,30].

A low CD4 cell count has been found to be a risk factor for LTFU among pre-ART patients in Durban, South Africa [31] and in Uganda [32]. Findings from these studies contradict our study finding where patients with higher CD4 cell counts were at an increased risk of LTFU (see Figure 1B). This could be due to methodological differences with respect to operational definitions and also differences between the study settings [31-33]. On the other hand, this study’s finding regarding the association between a less advanced disease stage and pre-ART LTFU was consistent with a study conducted in southern Ethiopia with similar operational definitions and study settings [6]. This finding was also supported by a study conducted in South Africa where the probability of returning to care reduced for patients with higher CD4 cell counts [34]. Part of the explanation for the increased risk of pre-ART LTFU among patients with better immune statuses or less advanced disease stages could result from patients’ self-assessment of feeling healthy and might have been more reluctant to be on pre-ART [9,35,36].

In this study, HIV status disclosure was found to be significantly associated with time until LTFU among pre-ART patients. Compared to those patients who disclosed their HIV status to their family members, those who did not disclose it to anyone had a nearly three times higher risk of LTFU (see Figure 1C). In one study, disclosure status did not show a significant association [37] and this could be due to differences in methodological variations. But, a study in Ethiopia indicated that HIV positive people experience both discriminatory exclusion and isolation from society, which further discourages disclosure of their HIV status. As a result, status disclosure and health-seeking behavior could be negatively affected. Similarly, among interviewed pre-ART patients from Addis Ababa, 50% had not disclosed their HIV status due to fear of rejection from household members, thus they attiritted from care [37]. Another systematic review of 34 studies supports the notion that HIV status disclosure can improve access to HIV/AIDS care and, therefore, the retention in pre-ART care [38]. Part of the underlying explanation for undisclosed HIV status can be fear of stigma and rejection from household members and the community at large [38,39].

### Limitations of the study

This study was not without limitations. The number of lost to follow-up patients might have been overestimated because patients might have been dying at home without reporting it. Using data with incomplete information might have also introduced bias.

## Conclusion

A high rate of LTFU among pre-ART patients was observed, with more than one-third of patients being lost to follow-up in the first year of pre-ART care. Analysis of predictor variables indicated that high LTFU rates were associated with no CPT at enrolment, a baseline CD4 cell count ≥350 cells/mm^3^, and not disclosing HIV status.

Close monitoring and improved tracking of patients is recommended during the early periods of pre-ART care. In addition, the preventive intervention should be scaled up in a manner that all CPT-eligible pre-ART patients get this intervention promptly. Treatment programs must make greater efforts to retain patients in care prior to ART initiation, particularly those who are in good health at enrollment, so that they can start ART as soon as they become eligible. Patients should also endeavor to avail services in their catchment area and increase timely communication with family members.

## Additional file

Additional file 1:
**Multilingual abstracts in the six official working languages of the United Nations.**

## Abbreviations

AIDS: Acquired immunodeficiency syndrome
ART: Antiretroviral therapy
ARV: Antiretroviral drug
CPT: Co-trimoxazole prophylactic therapy
HIV: Human immunodeficiency virus
JHU: John Hopkins University
LTFU: Loss to follow-up
MSH: Management science for health
PICT: Provider-initiated counseling and testing
PMTCT: Prevention of mother-to-child transmission
SSA: Sub-Saharan Africa
VCT: Voluntary counseling and testing
WHO: World Health Organization
