# Supplementary material for: When are patients lost to follow-up in pre-antiretroviral therapy care? a retrospective assessment of patients in an Ethiopian rural hospital
Source: Infect Dis Poverty. 2015 Jun 1;4:27. doi: 10.1186/s40249-015-0056-y (PMC4450642; doi:10.1186/s40249-015-0056-y)
Supplement: Additional file 1: — Multilingual abstracts in the six official working languages of the United Nations. [file 40249_2015_56_MOESM1_ESM.pdf]

Translation of the abstract into the six official working languages of the United Nations

متى يتم فقدان المرضى الذين يجب متابعتهم في فترة الرعاية خلال خضوعهم للعلاج الوقائي ضد فيروسات النسخ العكسي؟ تقييم للمرضى بأثر رجعي في مستشفى إثيوبي ريفي

تمرة شاوينو وديبيبي شاوينو

موجز

نبذة: ثمة قلق بشأن المعدلات المتزايدة لفقدان المتابعة الطويلة الأجل للمرضى (LTFU) بين المرضى الخاضعين للعلاج الوقائي ضد فيروسات النسخ العكسي (pre-ART) في إثيوبيا، والمعلومات المتوفرة المتعلقة بوقت فقدان المرضى لمتابعتهم في فترة خضوعهم لهذا العلاج الوقائي هي ضئيلة. قيّمت هذه الدراسة الوقت حيث يحدث فقدان المتابعة الطويلة الأجل للمرضى، إضافة إلى العوامل ذات الصلة بين الكبار المسجلين للحصول على الرعاية خلال خضوعهم لهذا العلاج في مستشفى إثيوبي ريفي.

الطرائق: جرى استعراض كافة البيانات الخاصة بالمرضى الكبار الخاضعين للعلاج الوقائي ضد فيروسات النسخ العكسي في مستشفى إقليم شوا ما بين 2010 و2013. واعتبر المرضى مفقودين لكي تتم متابعتهم في حال لم يحافظوا على المواعيد المحددة لفترة تتخطى الـ90 يوماً. استخدم نموذج تحوّف المخاطر النسبي للقاح كوكس (Cox) من أجل تقييم العوامل المرتبطة بالوقت لحين فقدان المتابعة الطويلة الأجل. كما استخدم جدول كابلان ماير (Kaplan-Meier) للوفيات والأحياء لمقارنة تجارب المرضى الذين يفقدون المتابعة الطويلة الأجل والمعزولين نتيجة مؤشرات ذات دلالة.

النتائج: تمت متابعة 626 مريضاً خاضعين للعلاج الوقائي ضد فيروسات النسخ العكسي لـ319.92 شخصاً – سنة من المراقبة (PYOs) بدءاً من تاريخ التسجيل وصولاً إلى نتائج العلاج الوقائي ضد فيروسات النسخ العكسي بمعدل عام للعلاج الوقائي ضد فيروسات النسخ العكسي بلغ 55.8 لكل 100 شخص – سنة. تمّ فقدان المتابعة الطويلة الأجل لـ 178 (28.4%) مريضاً خاضعاً للعلاج الوقائي ضد فيروسات النسخ العكسي، وحدثت نسبة 93% منها خلال فترة الستة أشهر الأولى، فبلغ متوسط فترة المتابعة 6.13 شهراً. وتضمنت المؤشرات المستقلة: عدم بدء الوقاية بواسطة الكوتريموكسازول (نسبة الخطر المعدل [AHR] = 1.77، نسبة نطاق الثقة [CI] بلغت 95% ، 1.12 – 2.79)، وبلغت القيمة القاعدية لعدد فرق مجموعة 4 [CD4] 350 خلية/مم^3 أو تخطتها (نسبة الخطر المعدل = 1.87، نسبة نطاق الثقة 95%، 1.02 – 3.45)، وحالة الإصابة بفيروس نقص المناعة البشرية (HIV) غير المعلنة (نسبة الخطر المعدل = 3.04، نسبة نطاق الثقة 95%، 2.07 – 4.45).

الاستنتاج: فُقدت نسبة كبيرة من متابعة المرضى الخاضعين للعلاج الوقائي ضد فيروسات النسخ العكسي. وشكل عدم بدء الوقاية بواسطة الكوتريموكسازول وخضوع المرضى للرعاية بلغت القيمة القاعدية لعدد فرق مجموعة خلاياهم 4 [CD4] 350 خلية/مم3 أو تخطتها ، إضافة إلى حالتهم غير المعلنة عن إصابتهم بفيروس نقص المناعة البشرية (HIV)، مؤشرات ذات دلالة لفقدان المتابعة الطويلة الأجل بين المرضى الخاضعين للعلاج الوقائي ضد فيروسات النسخ العكسي. لذا، يوصى بشدة مراقبة المرضى عن كثب خلال هذه الفترة إذ أن هؤلاء المرضى المحددة عوامل الخطر لديهم يستوجبون اهتماماً خاصاً.

Translated from English version into Arabic by Liliane Hatem, through

TRANSLATORS WITHOUT BORDERS

埃塞俄比亚乡村医院患者接受抗病毒治疗前失访时间及影响因素的回顾性研究

塔姆拉特 肖恩欧， 黛比 肖恩欧

摘要

引言：在埃塞俄比亚，接受抗病毒治疗前（pre-ART）的患者的失访率越来越高，引起人们担忧。但是关于pre-ART患者何时失访的资料甚少。本研究评估了在埃塞俄比亚乡村医院登记并接受pre-ART护理的成年患者的失访时间及影响因素。

方法：本文回顾了 2010 年至 2013 年间所有在卡法谢卡地区医院登记的成年 pre-ART 患者的资料。若患者超过 90 天未如约治疗，就被认定为失访。本文采用 Cox 比例风险回归模型来评估患者开始失访时间的相关因素，还采用 Kaplan-Meier 生存表比较了患者的失访经历，并用显著预测因子来区分。

结果：一共对 626 名 pre-ART 患者进行随访，追踪从登记住院开始到 pre-ART 结束，观察总人年数为 319.92 人年。总失访率为 55.8/100 人年。共有 178 名 pre-ART 失访患者(28.4%)。其中，有 93%的患者在前六个月内失访，随访时间中位数为 6.13 个月。独立预测因素包括：未以复方新诺明为开端疗法(校正危险比[AHR]=1.77, 95%置信区间[CI, 1.12-2.79])，基准 CD4 细胞数量≥350 个/ mm^3 (AHR=1.87, 95%CI, 1.02–3.45)，未查明 HIV 状况(AHR=3.04, 95%CI, 2.07–4.45)。

结论：研究结果表明，相当一部分 pre-ART 患者无法随访。pre-ART 失访患者的主要因素为：未以复方新诺明为开端疗法，基准 CD4 细胞数量≥350 个/ mm^{3}时和未查明 HIV 状况。因此，强烈建议在此期间对患者进行检测和随访，尤要关注具有失访危险因素的患者。

Translated from English version into Chinese by Chen Jin, edited by Yin Jian-hai, through

CHINESE CENTER FOR DISEASE CONTROL AND PREVENTION
NATIONAL INSTITUTE OF PARASITIC DISEASES

Quand les patients ne bénéficient-ils pas d'un suivi de soins d'une thérapie pré-antirétrovirale ? Evaluation rétrospective de patients dans un hôpital rural éthiopien.

Tamrat Shaweno and Debebe Shaweno

Sommaire

Contexte: En Ethiopie, l'augmentation du taux de l'absence de suivi (LTFU) parmi les patients en thérapie pré-antirétrovirale (pre-ART) est préoccupante. Il y a très peu d'informations disponibles concernant la période dans laquelle les patients pre-ART ne bénéficient pas d'un suivi dans le pays. Cette étude concerne la période à laquelle apparaît un LTFU ainsi que les facteurs associés parmi les adultes inscrits en soins pre-ART dans un hôpital rural éthiopien.

Méthodes: les données de tous les patients pre-ART adultes inscrits à l'hôpital zonal de Sheka entre 2010 et 2013 ont été examinées. Les patients étaient considérés comme ne bénéficiant pas d'un suivi s'ils ne se présentaient pas aux rendez-vous fixés pendant plus de 90 jours. Le modèle de la régression des risques proportionnels de Cox a été utilisé pour évaluer les facteurs associés à la période de temps jusqu'au LTFU. La table de survie Kaplan-Meier a été utilisée pour comparer les expériences LTFU des patients, séparés par des indicateurs significatifs.

Résultats: Un total de 626 patients pre-ART ont été suivis pour 319,92 personnes-années d'observation (PYO) depuis l'inscription jusqu'aux résultats pre-ART, avec un taux LTFU global de 55,8 pour 100 PYO. Un total de 178 (28,4%) de patients pre-ART n'ont pas bénéficié d'un suivi, dont 93% apparus dans les six premiers mois. Le temps de suivi médian était de 6,13 mois. Les indicateurs indépendants ont inclus: ne pas avoir été démarré sur une prophylaxie au co-trimoxazole (rapport des risques ajustés = adjusted hazard ratio [AHR] =1.77, 95% intervalle de confiance [CI], 1.12–2.79), un compte de base de référence CD4 de ou au-dessus 350 cellules/mm3 (AHR=1.87, 95%CI, 1.02–3.45), et un statut VIH non divulgué (AHR=3.04, 95%CI, 2.07–4.45).

Conclusion: Une proportion significative de patients pre-ART ne bénéficie pas d'un suivi. N'ayant pas été démarré sur une prophylaxie au co-trimoxazole, se présentant aux soins avec un compte de base de référence CD4 cellules ≥350 cellules/mm^3, et un statut VIH non divulgué, étaient des indicateurs significatifs de LTFU parmi les patients pre-ART. Par conséquent, une surveillance étroite et un suivi des patients pendant cette période sont fortement recommandés. Ces patients aux facteurs de risques identifiés méritent une attention spéciale.

Translated from English version into French by Ode Laforge, through

TRANSLATORS
WITHOUT BORDERS

Когда пациенты выбывают из наблюдения в ходе предварительной антиретровирусной терапии? Ретроспективная оценка пациентов в одной из сельских больниц Эфиопии

Тамрат Шоено и Дебебе Шоено

Реферат

История вопроса: Присутствует определенная озабоченность в связи с ростом случаев неявки для последующего наблюдения среди пациентов, проходящих курс предварительной антиретровирусной терапии в Эфиопии. Отсутствуют достаточные данные по времени выпадения пациентов, проходящих курс предварительной антиретровирусной терапии в стране. В данном исследовании производилась оценка периода, во время которого происходило выпадение из поля зрения взрослых пациентов, проходящих курс предварительной антиретровирусной терапии в одной из сельских больниц Эфиопии, а также ассоциированных факторов.

Методы: Были рассмотрены данные всех взрослых пациентов, проходящих курс предварительной антиретровирусной терапии в региональной больнице Sheka Zonal Hospital между 2010 и 2013 годом. Пациенты считались выпавшими из наблюдения, если они не являлись на запланированные встречи на протяжении более 90 дней. Для оценки факторов, ассоциированных с периодом, во время которого происходило выпадение пациентов из поля зрения, применялся регрессивный анализ пропорциональных рисков Кокса. Для сравнения опыта выпадения из наблюдения пациентов, выделенных на основе статистически достоверных прогностических факторов, использовалась таблица дожития Каплана Мейера .

Результаты: Всего наблюдению было подвергнуто 626 пациентов, проходящих курс предварительной антиретровирусной терапии, в объеме 319.92 пациенто-лет наблюдения (PYOs) с начала курса лечения до его завершения с общим коэффициентом выпадения из поля зрения, составляющим 55.8 на 100 пациенто-лет наблюдения. Всего выбыло из наблюдения 178 (28.4%) пациентов, проходящих курс предварительной антиретровирусной терапии, причем 93% случаев произошло в течение первых шести месяцев. Среднее время наблюдения составляло 6.13 месяцев. Независимые прогностические факторы включали: не был начат профилактический курс ко-тримоксазола

(скорректированное отношение рисков [AHR] =1.77, доверительный интервал для доверительной вероятности 0,95 [CI], 1.12–2.79), исходное число CD4-клеток в размере или выше 350 клеток/мм3 (AHR=1.87, 95%CI, 1.02–3.45) и необнаруженный ВИЧ статус (AHR=3.04, 95%CI, 2.07–4.45).
Выводы: Значительная часть пациентов, проходящих курс предварительной антиретровирусной терапии, выбывает из наблюдения. Значимыми прогностическими факторами выпадения из поля наблюдения для пациентов, проходящих курс предварительной антиретровирусной терапии являлись следующие: не был начат профилактический курс ко-тримоксазола, в начале курса терапии число CD4-клеток составляло ≥350 клеток/мм^3, необнаруженный ВИЧ статус. Следовательно, во время этого периода настоятельно рекомендуется осуществлять строгое наблюдение и отслеживание пациентов. Пациенты с выявленными факторами риска заслуживают особого внимания.

Translated from English version into Russian by Alena Hrybouskaya, through

TRANSLATORS
WITHOUT BORDERS

Cuándo se pierden los pacientes durante el seguimiento de la terapia antiretroviral previa (pre-TAR)?
Una evaluación retrospectiva de pacientes en un hospital rural en Ethiopia.

Tamrat Shaweno y Debebe Shaweno

Resumen de antecedentes: En Ethiopia es preocupante el aumento en la cantidad de pacientes que se pierden en el seguimiento de la terapia antiretroviral previa (pre-TAR). Hay poca información disponible con respecto al momento en que los pacientes en pre-TAR se pierden para el seguimiento del tratamiento en el país. Este estudio evaluó el momento cuándo la pérdida de los pacientes se produce así como también los factores asociados entre los adultos participando en pre-TAR en un hospital rural de Ethiopia.
Métodos: Fueron revisados los datos de todos los adultos en pre-TAR en el Hospital zonal de Sheka, entre 2010 y 2013. Los pacientes fueron considerados "perdidos para el seguimiento" si no asistían a las citas programadas durante más de 90 días. Se utilizó el modelo de regresión de riesgos proporcionales de Cox para evaluar los factores asociados con el tiempo hasta la pérdida en el seguimiento. La tabla de supervivencia de Kaplan-Meier se utilizó para comparar las experiencias de pérdida en el seguimiento de pacientes, segregados por factores predecibles significativos.
Resultados: Un total de 626 pacientes en pre-TAR fueron seguidos por 319.92 personas-años de observación (PAO) desde la inscripción hasta los resultados del pre-TAR, con una tasa global de pérdida en el seguimiento de 55,8 por cada 100 PAOs. Un total de 178 (28,4%) pacientes en pre-TAR se perdieron para el seguimiento, el 93% de las pérdidas se produjeron dentro de los primeros seis meses. El tiempo medio de seguimiento fue de 6,13 meses. La predicciones independientes incluyeron: no haber sido iniciado en la profilaxis con cotrimoxazol (razón de riesgo ajustada [RRA] = 1,77, 95% intervalo de confianza [IC], 1,12-2,79), un recuento de CD4 al inicio de/o por encima de 350 células/mm3 (RRA = 1,87, 95% IC, 1,02 a 3,45) y un estado de VIH no revelado (RRA = 3,04; IC 95%, 2,07-4,45).
Conclusión: Una proporción significativa de pacientes en pre-TAR se pierde durante el seguimiento. No haber sido iniciado en la profilaxis con cotrimoxazol, presentar en la atención una base de recuento de células CD4 ≥350/mm3 y un estado no revelado de VIH fueron predicciones significativas de pérdida en el seguimiento entre los pacientes en pre-TAR.
Por lo tanto, es muy recomendable una estrecha vigilancia y seguimiento de los pacientes durante este periodo. Los pacientes con factores de riesgo identificados se merecen una atención especial.

Translated from English version into Spanish by patriciacassoni, through

TRANSLATORS
WITHOUT BORDERS
